# Dual Mobility Versus Conventional Total Hip Arthroplasty for Femoral Neck Fractures: A Systematic Review and Meta-Analysis Comparing Dislocation and Re-operation Rates

**DOI:** 10.7759/cureus.85504

**Published:** 2025-06-07

**Authors:** Ashwani Nugur, Ashok Goel

**Affiliations:** 1 Trauma and Orthopaedics, Ysbyty Gwynedd Hospital, Bangor, GBR

**Keywords:** dislocation, dual-mobility cup total hip replacement, femoral neck fracture, re-operation, total hip replacement/arthroplasty

## Abstract

Dual mobility (DM) implants have gained increasing attention in revision total hip arthroplasty and among patients at high risk of dislocation due to their enhanced stability. This systematic review and meta-analysis aimed to assess the viability of dual mobility total hip replacement (DM-THR) by comparing dislocation and re-operation rates between patients treated with DM-THR and those treated with conventional total hip replacement (C-THR) for displaced femoral neck fractures. A systematic review of the published literature was conducted using PubMed (MEDLINE), PsycINFO, Cochrane databases, and Google Scholar to identify studies comparing DM-THR with C-THR, in accordance with the Preferred Reporting Items for Systematic Reviews and Meta-Analyses (PRISMA) guidelines. Nine studies, comprising a total of 9,816 patients with femoral neck fractures, were analysed. Of these, 7,197 patients underwent C-THR, while 2,619 received DM-THR. The cumulative dislocation rate was 1.35%, with a dislocation rate of 1.5% for C-THR and 0.8% for DM-THR. The cumulative reoperation rate was 3.61%, with reoperation rates of 3.87% for C-THR and 2.9% for DM-THR. Forest plots from the meta-analysis favoured DM-THR over C-THR (P < 0.05, 95% confidence interval (CI)) in terms of dislocation rate; however, no significant difference was observed in the re-operation rate. Further large-scale, high-quality randomised controlled trials with long-term follow-up and cost analysis are required to establish DM-THR as a viable and effective option for femoral neck fractures.

## Introduction and background

Femoral neck fractures are the most frequent injury among the elderly population globally [1]. Each year, approximately 1.6 million hip fractures occur worldwide, with estimates suggesting this figure could rise to between 4.5 million and 6.5 million by 2050 [2]. In the United Kingdom, around 76,000 patients are admitted annually with femoral neck fractures, with half of these cases involving displaced intracapsular fractures [3].

Hemiarthroplasty (HA) and total hip replacement (THR) are widely accepted treatments for femoral neck fractures. While THR provides better functional outcomes and higher patient satisfaction [4-7], it is preferable for those with good life expectancy [3]. HA is often chosen as THR carries a higher dislocation risk [8-10]. Dislocation rates after THR for femoral neck fractures can reach 22%, averaging around 5%, compared to 0.12-16% in primary THRs for other indications [11-13]. This elevated risk is partly due to soft tissue damage from the fracture itself [14-17]. Risk-reduction strategies include using femoral heads larger than 28 mm, anterior or lateral surgical approaches [18-20], and dual mobility (DM) implants, which have been shown to lower dislocation rates [21-23].

The concept of dual mobility total hip replacement (DM-THR) was first developed by French professor Gilles Bousquet and engineered by André Rambert in 1974 [24]. The DM construct consists of a metal acetabular shell housing a polyethylene hemisphere liner, into which the femoral head fits, creating two articulating surfaces. Primary movement occurs between the femoral head and liner, and secondary movement occurs between the liner and shell. This dual articulation increases range of motion and jump distance, thereby reducing the risk of dislocation. Research has demonstrated that DM cups significantly reduce the dislocation risk in revision THRs and are also cost-effective compared to conventional total hip replacements (C-THRs) [25]. Positive outcomes from revisions have prompted an increasing use of DM prostheses in primary hip arthroplasty, which has similarly shown a significant reduction in dislocation rates [26]. The primary objective of this study is to compare the incidence of dislocation and the rate of re-operation between DM cups and conventional single mobility cups in THRs for femoral neck fractures.

## Review

Materials and methods

Study Objectives

The objective is to compare the incidence of dislocations and the rate of reoperations between THR with DM and conventional single mobility cups. Data for this analysis were extracted from relevant studies, and a meta-analysis was conducted using RevMan software (version 5.0; The Cochrane Collaboration, Oxford, UK) [27].

Inclusion Criteria

This study included comparative studies involving patients who underwent DM-THR for femoral neck fractures. We reviewed articles published from 2000 to 2023.

Exclusion Criteria

Non-comparative studies were excluded from this review. Additionally, we excluded studies that focused on THR for conditions other than femoral neck fractures. Case reports, case series, articles consisting solely of technical descriptions, studies with unclear outcomes, and studies related to revision THRs were also excluded.

Search Strategy

**Figure 1:**
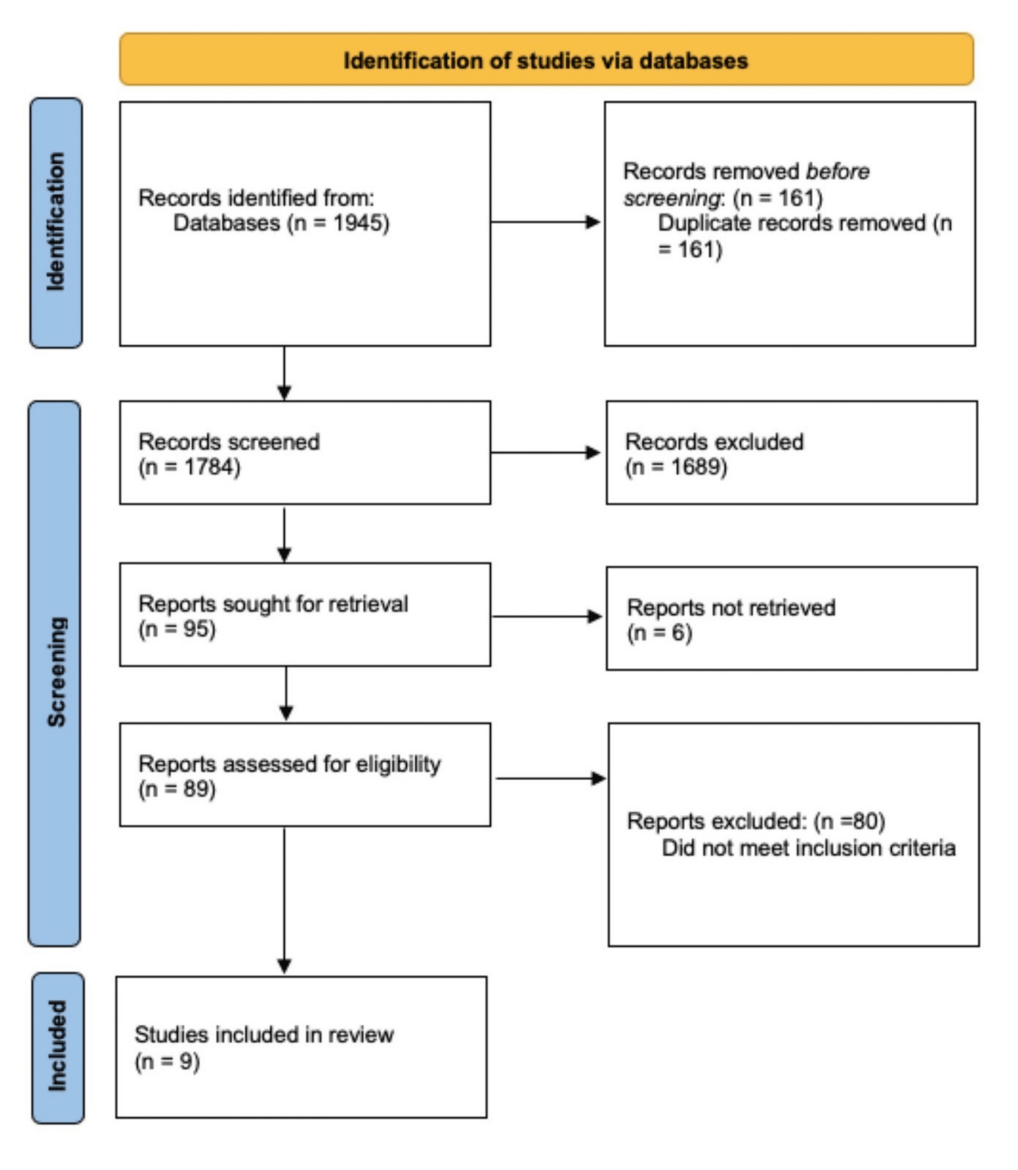
PRISMA flow diagram for the searching and identification of included studies PRISMA: Preferred Reporting Items for Systematic Reviews and Meta-Analyses

The article selection process followed the Preferred Reporting Items for Systematic Reviews and Meta-Analyses (PRISMA) guidelines (Figure 1) [28]. The databases used for this review included PubMed (MEDLINE), PsycINFO, Cochrane databases, and Google Scholar to identify relevant articles. To obtain the desired references from these databases, compound keywords were used, such as “Total hip arthroplasty and neck of femur”, “Dual mobility acetabular total hip replacement and neck of femur”. Primary outcome measures were incorporated into the search using keyword combinations such as “Femoral neck fracture and dual mobility hip arthroplasty and dislocation”, “Reoperation”, “Femoral neck fracture and conventional total hip arthroplasty and dislocation”, and “Reoperation”.

Two studies from the Nordic Joint Registry had overlapping patient data, and only one study was included. Ultimately, nine studies comparing the outcomes of DM and C-THRs in femoral neck fractures were included in the systematic review.

Study Selection

Relevant studies were selected through a two-step independent search process. Initially, the available titles and abstracts identified in the searches were analysed to determine their eligibility for inclusion in the review. In the second phase, full-text articles were evaluated in detail. Any conflicts between the two reviewers were resolved through discussion and agreement. The selected papers were assessed for quality using the Critical Appraisal Skills Programme (CASP) checklist [29].

Risk of Bias

Nine studies included a total of 9,816 patients with femoral neck fractures, of whom 7,197 were treated with C-THR and 2,619 with DM-THR. Among these, one study was a randomised controlled trial, one was a prospective cohort study, and seven were retrospective studies, including one from a joint registry. A risk assessment of the studies was conducted, as shown in Table 1.

**Table 1:** Risk of bias assessment of individual studies

Author	Selection bias		Performance bias	Detection bias	Attrition bias	Reporting bias	Other bias
	Random sequence generation	Allocation concealment	Blinding of participants	Blinding of outcome bias	Incomplete outcome data	Selective reporting	
Agarwala et al., 2021 [30]	High risk	High risk	High risk	High risk	Low risk	Low risk	Low risk
Alberio et al., 2021 [31]	High risk	High risk	High risk	High risk	Low risk	Low risk	Low risk
Goru et al., 2022 [32]	High risk	High risk	High risk	High risk	Moderate	Low risk	Low risk
Ochi et al., 2017 [33]	High risk	High risk	High risk	High risk	Low risk	Low risk	Low risk
Rashed et al., 2021 [34]	Low risk	Low risk	Low risk	Moderate	Low risk	Low risk	Low risk
Rogmark and Nauclér, 2022 [35]	High risk	High risk	High risk	High risk	Low risk	Low risk	Low risk
Sadozai et al., 2021 [36]	High risk	High risk	High risk	High risk	Moderate	Low risk	Low risk
Tarasevicius et al., 2010 [37]	High risk	High risk	High risk	High risk	Low risk	Moderate	Moderate
Zagorov et al., 2018 [38]	High risk	High risk	High risk	High risk	Low risk	Low risk	Low risk

Results

The demographics and extracted data are presented in Tables 2-3.

**Table 2:** Study design and participant demographics DM-THR: dual mobility total hip replacement; C-THR: conventional total hip replacement

Author	Design	Study period	DM-THR	C-THR	Mean age (years)		Mean follow-up (months)		
					DM-THR	C-THR	DM-THR	C-THR	
Agarwala et al., 2021 [30]	Prospective cohort	2018-2019	52	51	75.58	73.37	12		12
Alberio et al., 2021 [31]	Retrospective	2017-2019	24	24	77.03	78.35	23		23
Goru et al., 2022 [32]	Retrospective + prospective observational	2017-2020	24	51	NA	NA	12		12
Ochi et al., 2017 [33]	Retrospective	2009-2015	33	36	80	75.2	15.8		17.9
Rashed et al., 2021 [34]	Randomised controlled trial	2014-2015	31	31	66.38	68	12		12
Rogmark and Nauclér, 2022 [35]	Retrospective	2005-2019	2242	6726	76	75.2	60		60
Sadozai et al., 2021 [36]	Retrospective	2012-2018	127	195	70	70	37		37
Tarasevicius et al., 2010 [37]	Retrospective	2004-2008	42	56	75	74	12		12
Zagorov et al., 2018 [38]	Retrospective	2012-2017	44	27	73.4	70	29.7		36.6

**Table 3:** Outcomes of individual studies C-THR: conventional total hip replacement; DM-THR: dual mobility total hip replacement; MHA: Modified Hardinge approach; PA: posterior approach; DAA: direct anterior approach; LA: lateral approach; SPAIRE: Sparing Piriformis and Internus, Repair Externus; HHS: Harris hip score; WOMAC: The Western Ontario and McMaster Universities Arthritis Index

Author	Surgical approach, n (%)		Dislocation, n (%)		Re-operation, n (%)		Functional outcome		Mortality at 1 year	
	C-THR	DM-THR	C-THR	DM-THR	C-THR	DM-THR	C-THR	DM-THR	C-THR	DM-THR
Agarwala et al., 2021 [30]	MHA	MHA	None	None	None	None	HHS 76.3	HHS 87.1	None	None
Alberio et al., 2021 [31]	PA	PA	2 (8.3%)	None	None	None	WOMAC 4.94	WOMAC 7.58	8.3%	8.3%
Goru et al., 2022 [32]	MHA 29, PA 22	MHA 0, PA 24	6 (11.7%)	1 (4.1%), Intraprosthetic	None	None	NA	NA	NA	NA
Ochi et al., 2017 [33]	DAA	DAA	None	None	None	None	Walking ability - No difference	Walking ability - No difference	2%	2%
Rashed and Nauclér, 2021 [34]	PA (100%)	PA (100%)	None	None	None	None	HHS 86.62	HHS 92.8	None	None
Rogmark et al., 2022 [35]	PA 2492, LA 4234	PA 1114, LA 1128	PA 54 (2.2%), LA 29 (0.7%)	PA 14 (1.3%), LA 4 (0.4%)	275 (4.1%)	76 (3.4%)	NA	NA	NA	NA
Sadozai et al., 2021 [36]	NA	NA	10 (5.13%)	2 (1.57%)	None	None	NA	NA	NA	NA
Tarasevicius et al., 2010 [37]	PA	PA	8 (14%)	None	3 (5.3%)	None	NA	NA	NA	NA
Zagorov et al., 2028 [38]	SPAIRE	SPAIRE	3 (11.1%)	None	1 (3.7%)	None	NA	NA	3.7%	7.1%

Dislocation and Reoperation

**Figure 2:**
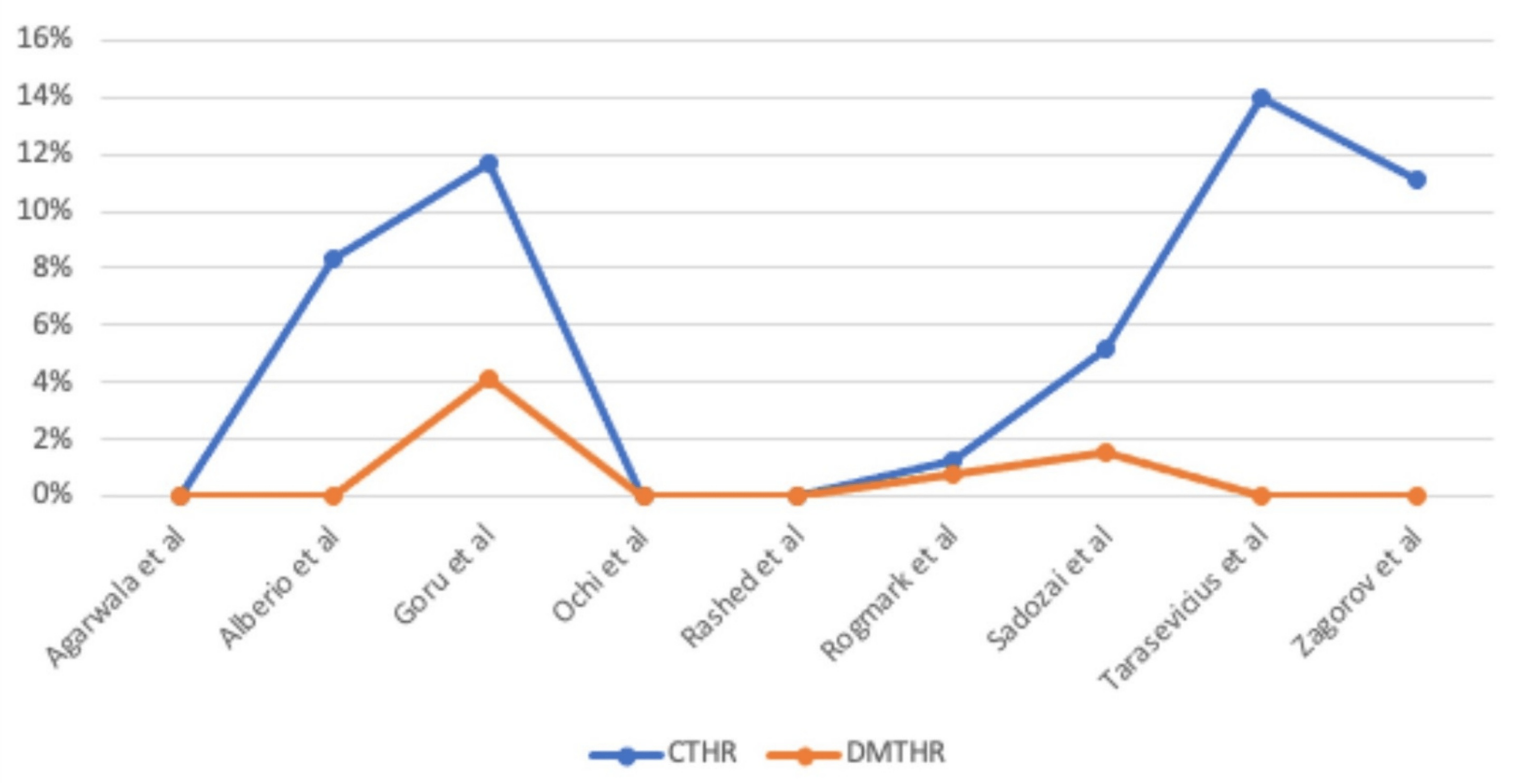
Graph illustrating the dislocation rates across studies CTHR: conventional total hip replacement; DMTHR: dual mobility total hip replacement Studies included: Agarwala et al. [30], Alberio et al. [31], Goru et al. [32], Ochi et al. [33], Rashed et al. [34], Rogmark and Nauclér [35], Sadozai et al. [36], Tarasevicius et al. [37], Zagorov et al. [38].

The cumulative dislocation rate identified was 1.35%, with a dislocation rate of 1.5% for C-THR and 0.8% for DM-THR (Figure 2).

Studies by Agarwala et al. [30], Ochi et al. [33], and Rashed et al. [34] showed no difference in dislocation rates between the groups, whereas studies by Alberio et al. [31], Goru et al. [32], Rogmark and Nauclér [35], Sadozai et al. [36], Tarasevicius et al. [37], and Zagorov et al. [38] reported a lower dislocation rate with DM-THR.

**Figure 3:**
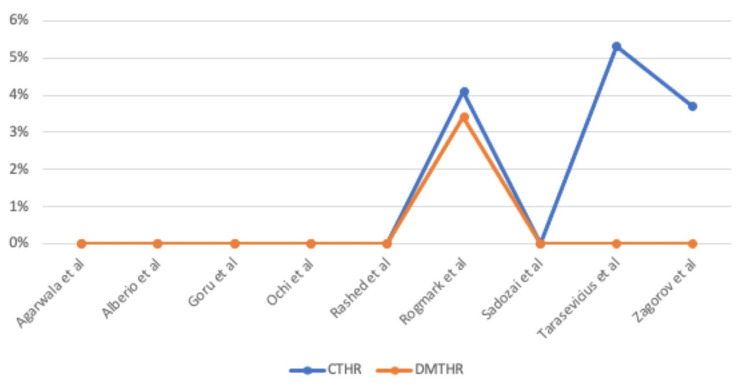
Graph illustrating the re-operation rates across studies CTHR: conventional total hip replacement; DMTHR: dual mobility total hip replacement Studies included: Agarwala et al. [30], Alberio et al. [31], Goru et al. [32], Ochi et al. [33], Rashed et al. [34], Rogmark and Nauclér [35], Sadozai et al. [36], Tarasevicius et al. [37], Zagorov et al. [38].

The cumulative reoperation rate was 3.61%, with a reoperation rate of 3.87% for THR with a single mobility cup and 2.9% for DM-THR (Figure 3).

Six out of nine studies [30-34,36] showed no difference in re-operation rates between the groups, whereas the remaining three studies by Rogmark et al. [35], Tarasevicius et al. [37], and Zagorov et al. [38] reported a lower re-operation rate with DM-THR.

Meta-Analysis

In the forest plots, the DM option was treated as the experimental group, while the C-THR option was treated as the control group. The corresponding forest plot is shown below.

**Figure 4:**
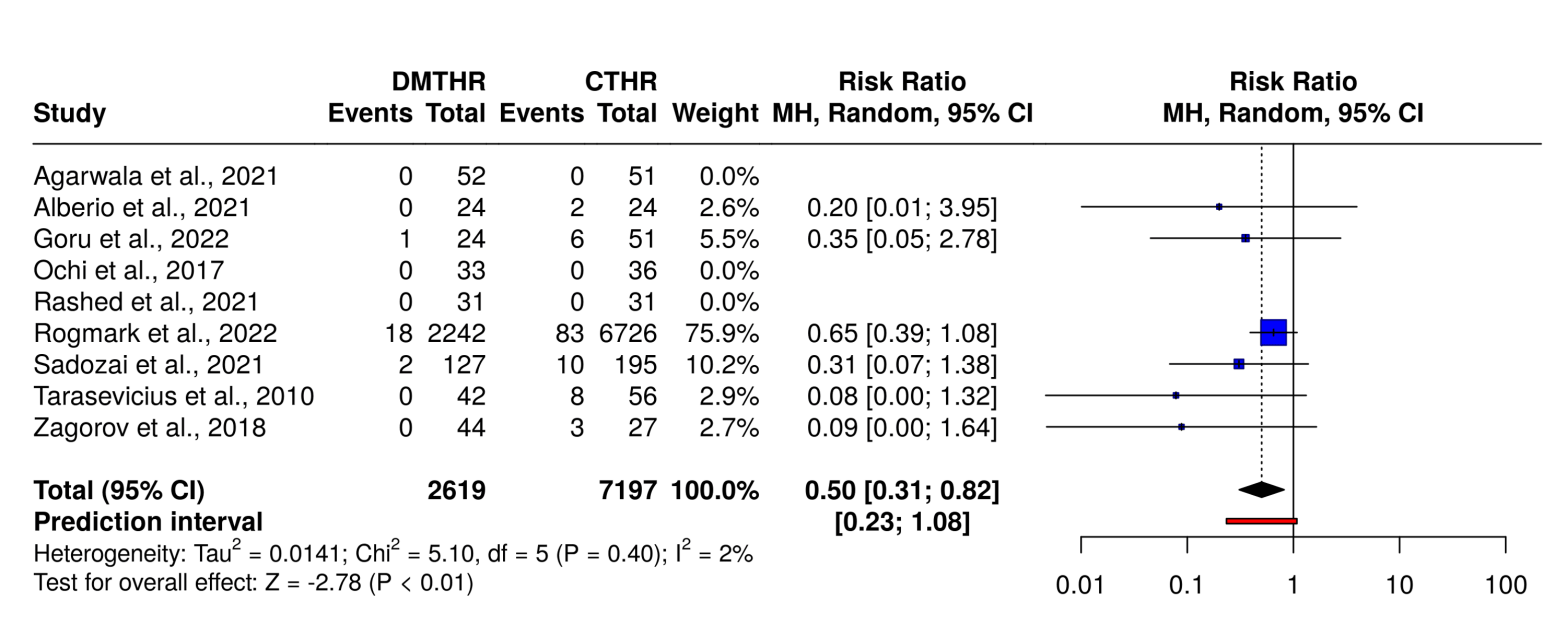
Forest plot comparing dislocation rates DMTHR: dual mobility total hip replacement; CTHR: conventional total hip replacement Studies included: Agarwala et al. [30], Alberio et al. [31], Goru et al. [32], Ochi et al. [33], Rashed et al. [34], Rogmark and Nauclér [35], Sadozai et al. [36], Tarasevicius et al. [37], Zagorov et al. [38].

In Figure 4, meta-analysis performed using a random-effects model with the Mantel-Haenszel method to compare the risk ratio revealed a statistical difference. The summarised risk ratio is 0.5, with a 95% confidence interval of 0.31-0.82. The analysis for the overall effect indicates significance at p < 0.05. No significant heterogeneity was observed, suggesting that effect sizes across studies are consistent in both magnitude and direction.

**Figure 5:**
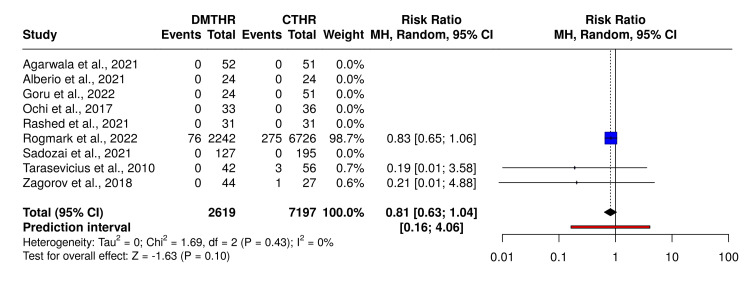
Forest plot comparing re-operation rates DMTHR: dual mobility total hip replacement; CTHR: conventional total hip replacement Studies included: Agarwala et al. [30], Alberio et al. [31], Goru et al. [32], Ochi et al. [33], Rashed et al. [34], Rogmark and Nauclér [35], Sadozai et al. [36], Tarasevicius et al. [37], Zagorov et al. [38].

In Figure 5, based on the analysis performed using a random-effects model with the Mantel-Haenszel method to compare the risk ratio, no statistical difference was found between the two cohorts. The overall risk ratio is 0.81, with a 95% confidence interval of 0.63-1.04. The test for overall effect does not indicate a significant effect. No notable variability was detected, suggesting that the effect sizes across cohorts remained consistent in both scale and direction.

Functional Outcomes

Four out of nine studies reported on functional outcomes. In the study by Agarwala et al., the Harris Hip Scores (HHS) were significantly higher in the DM-THR group, with scores of 76.37 at three months and 87.02 at one year, compared to the C-THR group, which had scores of 65.65 at three months and 72.96 at one year. The study concluded that patients treated with DM cups demonstrated a greater range of motion and improved function [30].

In the study by Alberio et al., the overall Western Ontario and McMaster Universities Arthritis Index (WOMAC) score was lower in the DM-THR group (4.94) compared to the C-THR group (7.58), though the p-value was 0.41, indicating no statistical significance [31].

In the study by Ochi et al., walking ability was assessed as a functional outcome, and no difference was observed between the DM-THR and C-THR groups [33].

In a randomised controlled trial by Rashed et al., the DM-THR group had higher HHS scores at 4, 6, and 12 months, and their range of motion was statistically superior to that of the C-THR group (p = 0.001). The study concluded that DM-THR offers a better range of motion and improved functional outcomes compared to traditional THR [34].

Discussion

Femoral neck fractures are among the most common osteoporotic fractures in the elderly and are associated with substantial morbidity and mortality. This review investigated the effectiveness of DM-THR in reducing dislocation and re-operation rates in patients undergoing THR for femoral neck fractures.

Evidence indicates that THR using a DM cup produces positive outcomes for elderly patients. In a prospective multicenter study conducted by Adam et al. [39] in France, 214 patients with displaced femoral neck fractures were treated with DM-THR. They reported that three patients (1.4%) had dislocations that were reduced with closed manipulation, and none of the patients had any recurrence. They have mentioned that all three dislocations were of the large articulation of DM between the metallic shell and polyethylene liner, and none of them had intraprosthetic dislocations (IPDs). They suggest that DM cups are a valuable option for elderly patients with femoral neck fractures requiring THR.

A study by Nich et al. [40] involved 82 patients (83 hips) aged over 75 years who underwent THR using a DM cup with a posterolateral approach due to intracapsular neck of femur fractures. Clinical data were collected from 45 patients, with a mean follow-up period of 23.8 months. The study reported positive functional outcomes; however, three patients experienced postoperative dislocations, all of which were attributed to technical errors. Among these, two were dislocations of the large articulation (4.4%), and one was an IPD (2.2%). The authors concluded that while performing THR with a DM cup is technically demanding, it can help prevent dislocations and improve functional outcomes for elderly patients with femoral neck fractures.

Furthermore, an analysis of 78,098 THRs from the Swedish Joint Registry, conducted by Hailer et al., shows that after a mean follow-up of 2.7 years (ranging from 0 to 6 years), 399 hips (0.5%) had to be revised due to dislocation. They found that using 22-mm femoral heads posed a higher risk of revision compared to using 28-mm heads (relative risk = 2.0, confidence interval: 1.2-3.3). Notably, only 1 out of 287 DM cups required revision due to dislocation. This study indicates that the risk of dislocation can be reduced by using DM cups or femoral heads with a diameter larger than 28 mm, although this observation was not statistically significant [41].

A recent meta-analysis published in 2022 examined data from international joint registries to assess the revision rate of THRs for femoral neck fractures, comparing DM cups with C-THRs. Data were retrieved from six members of the International Society of Arthroplasty Registries, including the United Kingdom, Australia, the Netherlands, the United States, and Sweden, covering the period from 2002 to 2019. Data from 15,024 DM-THRs and 97,200 C-THRs were available for assessment. The reported revision rates were similar, at 4.7% (95% CI) for DM-THR and 4.3% (95% CI) for C-THR. However, in the DM-THR group, the primary cause of revision was infection. The authors concluded that the risk of revision surgery is not specifically reduced with DM-THR [42].

Despite their advantages, DM cups are associated with certain drawbacks. Potential complications include (1) risk of IPD: Although modern designs have reduced this risk, IPD remains a unique complication of DM-THR and may require open reduction if it occurs; (2) Potential for accelerated wear: The presence of two articulating surfaces raises concerns about polyethylene wear. However, recent studies suggest that wear rates are not significantly higher than those of conventional implants; (3) Cup loosening and mechanical issues: Proper surgical technique is critical for achieving optimal outcomes. Poor positioning can lead to complications such as instability or impingement. Cases have been reported of cup loosening or iliopsoas impingement due to the design or placement of the DM cup; (4) Debatable long-term survivorship: There is ongoing debate regarding whether DM-THR has a shorter lifespan compared to C-THR due to its more complex mechanics. However, evidence from modern designs suggests promising long-term survival rates [11,43,44].

THRs with DM cups have potential advantages of reduced dislocation rates and better functional outcomes; however, additional research is required before recommending a THR after femoral neck fracture, in order to evaluate the potential costs and risks associated with a more complex procedure.

Limitations

The limitations of this systematic review, which includes nine studies, arise from the fact that there is only one randomised controlled trial, which has a small patient sample and a follow-up period of just one year. Although the trial received ethical approval and patients were randomised, neither patient blinding nor assessor blinding is clearly stated.

Additionally, the review includes only one prospective cohort study, which also involved a small number of patients with a 12-month follow-up. The remaining seven studies are retrospective, with one study based on data from a joint registry. The joint registry data play a significant role in estimating the intended outcomes of this review, as they cover a large patient population with five years of follow-up, documenting dislocation rates, revision rates, and one-year mortality. The other six retrospective studies had limited patient numbers in both groups but reported on outcomes such as dislocation rates, surgical approaches, and functional scores.

## Conclusions

The findings demonstrated a statistically significant reduction in dislocation rates in favour of DM-THR when compared to C-THR, indicating a clear advantage in terms of postoperative stability. Despite this benefit, no significant difference was noted in re-operation rates between the two groups, suggesting that the improved stability did not necessarily translate into a reduced need for subsequent surgical intervention. Furthermore, emerging evidence from studies points toward improved functional outcomes with DM implants, including enhanced range of motion and greater patient-reported satisfaction, which may contribute to better overall quality of life postoperatively.

However, despite these promising results, the current literature remains limited, and there is an associated learning curve to this operative technique to achieve an optimum outcome. To establish DM-THR as a viable and effective option for treating femoral neck fractures, there is a need for large-scale, high-quality randomised controlled trials. These studies should incorporate long-term follow-up to assess implant longevity and patient outcomes, as well as comprehensive cost-effectiveness analyses to determine the economic viability of this approach in routine clinical practice.
